# Canadian Physicians’ Use of Ultrasound in Spasticity Treatment: A National Cross-Sectional Survey

**DOI:** 10.1016/j.arrct.2024.100353

**Published:** 2024-06-24

**Authors:** Fraser MacRae, Ève Boissonnault, Alto Lo, Heather Finlayson, Paul Winston, Omar Khan, Heather Dow, Farris Kassam, Rajiv Reebye

**Affiliations:** a Faculty of Health Sciences, Western University, London, Ontario, Canada; b Vancouver Island Health Authority, Victoria, British Columbia, Canada; c Canadian Advances in Neuro-Orthopedics for Spasticity Consortium (CANOSC), Kingston, Ontario, Canada; d Faculty of Medicine, Université de Montréal, Montréal, Quebec, Canada; e Physical Medicine and Rehabilitation, University of Alberta, Edmonton, Alberta, Canada; f Physical Medicine and Rehabilitation, University of British Columbia, Vancouver, British Columbia, Canada; g Hotel Dieu Shaver Health and Rehabilitation Centre, St. Catharines, Ontario, Canada

**Keywords:** Rehabilitation, Spasticity, Ultrasound

## Abstract

**Objective:**

To identify potential barriers and obstacles preventing clinicians from adopting ultrasound for spasticity management.

**Design:**

A prospective, cross-sectional national survey.

**Setting:**

Web-based platform.

**Participants:**

Thirty-six physicians and surgeons from across Canada.

**Interventions:**

Survey completion.

**Main Outcome Measures:**

The use of ultrasound in clinical spasticity practice, perceived barriers, and risks associated with its implementation.

**Results:**

In total, 36 Canadian physicians and surgeons responded. A total of 91% reported using the US in their practice. Nearly all of them used ultrasonography (US) to guide injections and reported using more than 1 guidance technique for their injections. Less than half of the survey respondents reported using the US for muscle architecture assessment or longitudinal evaluation of muscle echo intensity. A total of 47% of survey respondents reported that they believe there are disadvantages associated with US use in spasticity practice. Disadvantages included increased time requirements resulting in discomfort for the injector and patient, the risk of infection after the procedure, and the risk of needle-stick injury. The most important barrier identified was the increased time demands of US compared with other guidance techniques. Other barriers included a lack of feedback on identifying a spastic muscle compared with electrical guidance techniques, a lack of additional remuneration to complete injections under ultrasound guidance, and a lack of adequate training.

**Conclusions:**

Future educational efforts should address clinicians’ lack of familiarity with US purposes outside of injection guidance. This survey has highlighted the need for a curriculum shift in spasticity education to improve physician's scanning and injection technique, to address concerns about increased time requirements for injecting under ultrasound guidance and to address perceived disadvantages from clinicians.

Spasticity can occur after an upper motor neuron injury and presents as involuntary length- and velocity-dependent decreases in range of motion.^1^, ^2^, ^3^ Focal spasticity is managed routinely with botulinum toxin (BoNT) injections. BoNT functions as an acetylcholine inhibitor, acting on the presynaptic terminal in the neuromuscular junction and preventing the release of the excitatory neurotransmitter.^4^ Traditionally, BoNT is injected into the selected muscles, relying on anatomical landmarks for localization. However, there is level 1 evidence in support of guidance techniques such as ultrasound, electromyography, or electrical stimulation to localize target structures more precisely and improve patient outcomes.^5^

Ultrasonography (US) has recently become popular among physicians who inject BoNT for spasticity. US guidance has demonstrated noninferiority to electrical stimulation for BoNT injections for spasticity.^6^^,^^7^ Diagnostic nerve blocks, an emerging screening tool to help clinicians correctly identify spastic muscles, are recommended to be performed under US guidance.^8^^,^^9^ US provides additional information compared with electrical stimulation or electromyographic guidance techniques. US may improve safety by providing an image of the muscle and vasculature relative to the needle, likely reducing the likelihood of blood vessel puncture. Further, the US allows for an assessment of muscle size and echogenicity.^5^ As muscle atrophy occurs, palpation-based and even electromyograph-guided approaches may become more challenging. Regarding echogenicity, causes are unclear, but 1 study by Picelli et al^10^ showed that spastic gastrocnemius muscle echo intensity was directly associated with the modified Ashworth scale score and inversely correlated with muscle thickness, compound muscle action potential amplitude, and ankle passive range of motion.^10^ US evaluation of spastic muscles may therefore be a means of objectively assessing muscle health. The Heckmatt scale is a semiquantitative tool for assessing muscle architecture, where muscles with higher echogenicity score higher.^11^ The effects of spasticity treatment have been shown to diminish with increased muscle echo intensity.^12^, ^13^, ^14^, ^15^ Patients with higher Heckmatt scores experience less successful functional rehabilitation compared with those with lower scores when treated with the same BoNT protocols.^12^ Although the Heckmatt scale has not been validated for spasticity, the modified Heckmatt scale better differentiates between grades and has been shown to be valid and reliable in assessing pathologic muscle changes in patients with spasticity.^11^

US is an emerging technique in physical medicine and rehabilitation with the potential to improve the quality of care for patients with spasticity. The purpose of the present study was to probe the use of US guidance by Canadian physicians who treat spasticity to identify potential factors that may prevent its use in clinical practice.

## Methods

This study was approved by the local research ethics board. Informed consent was obtained from all participants who completed the survey. This study conforms to all Strengthening the Reporting of Observational studies in Epidemiology reporting guidelines for observational studies and reports information accordingly.

### Study Design

A national survey was designed for Canadian physicians who manage and treat patients with spasticity. The survey was hosted using the web-based platform Alchemer (www.alchemer.com). The survey contained a total of 49 questions in either an open-ended or multiple-choice format. The survey is included in the supplemental material (available online only at http://www.archives-pmr.org/).

### Canadian Advances in Neuro-Orthopedics for Spasticity Consortium

Canadian Advances in Neuro-Orthopedics for Spasticity Consortium (CANOSC) is a Canadian national organization and a part of the Canadian Association of Physical Medicine & Rehabilitation. It is the largest Canadian organization focused on advancing the field of spasticity through education and collaboration. CANOSC has 167 active Canadian members involved in the treatment of spasticity; 113 of whom are Physical Medicine and Rehabilitation (PM&R) specialists. The remainder of the members are orthopedic surgeons, pediatricians, neurosurgeons, plastic surgeons, neurologists, and allied health professionals. In 2019, a survey by the Canadian Medical Association found that there were approximately 500 PM&R specialists practicing in Canada; therefore, an estimated 22.6% of all practicing Canadian PM&R specialists are members of CANOSC, making CANOSC a suitable organization to examine US utilization in spasticity management.^16^

### Recruitment

**Fig 1:**
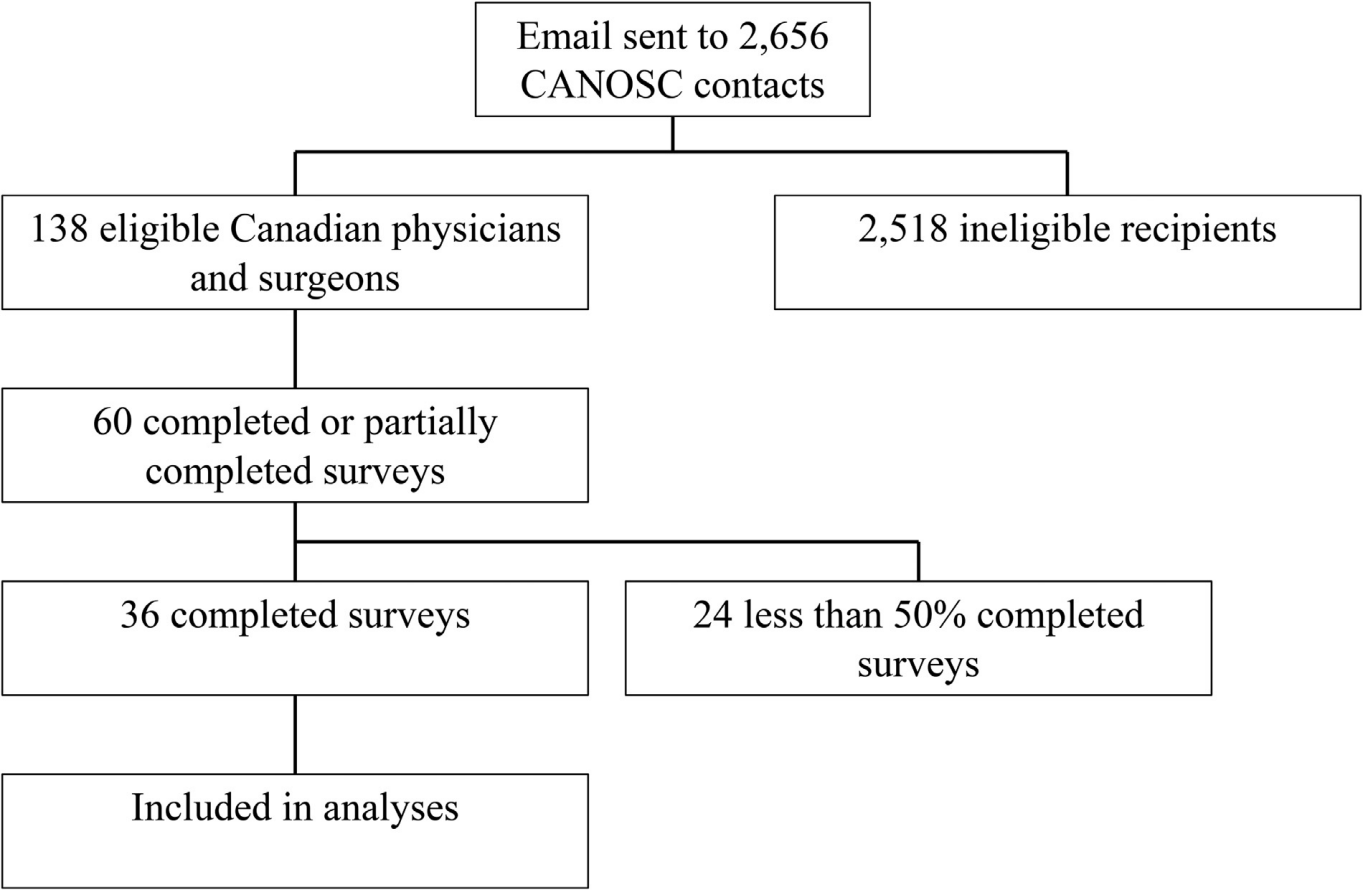
Recruitment process. A recruitment email was sent to the CANOSC mailing list. All international contacts and Canadian allied health professionals were excluded. A total of 138 eligible Canadian physicians and surgeons received the survey. Of them, 67 viewed the survey and 34 completed it in its entirety.

An email was sent to all CANOSC members (2656 total members, 167 Canadian members, including 138 physicians and surgeons). Refer to figure 1 for a summary of the recruitment process. The email contained details about the questionnaire, privacy information, a request for informed consent, and a link to the questionnaire. The survey was open for responses for 6 months, with email reminders sent out to contacts on 4 separate occasions during that period. Participants were aware that there was no compensation or incentive provided for survey completion.

## Results

Out of 138 eligible recipients, 36 Canadian physicians from 8 provinces and one territory completed the entire survey, for a response rate of 26.09%. Table 1 presents the demographic data of the respondents. Figure 1 describes the entire recruitment process. In total, 32 survey respondents indicated that they use a US machine in their spasticity practice. Two of the 3 participants who do not use a US machine in their spasticity practice indicated that their medical center or clinic does not have a US machine available for their use.

**Table 1:** Survey respondent demographic information

Demographic caracteristics	Demographic subgroups	Number or participants (n=36)
Licencing status	Fully licensed/attending physician	35
	Fellow	1
Medical specialty	Physical medicine and rehabilitation	30
	Orthopedic surgery	6
Gender	Female	22
	Male	14
Province or territory of practice	Alberta	3
	British Columbia	6
	Manitoba	1
	New Brunswick	1
	Ontario	9
	Prince Edward Island	6
	Quebec	5
	Saskatchewan	4
	Yukon	1

**Fig 2:**
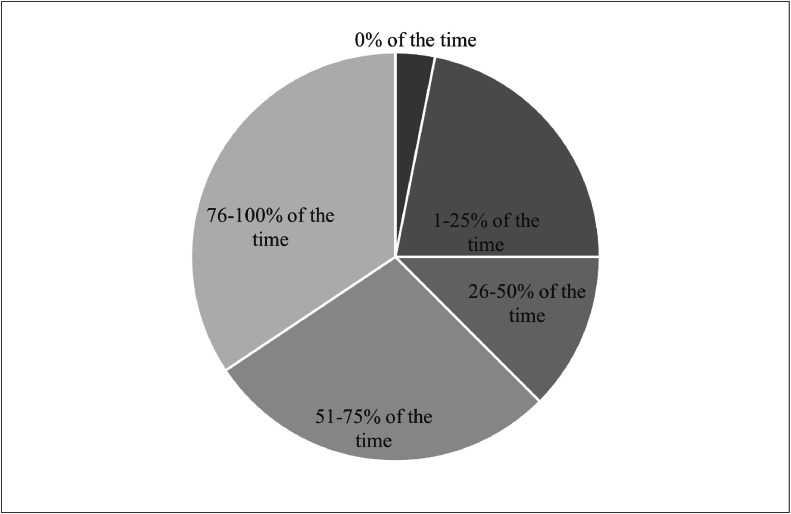
Proportion of spasticity injections completed under US guidance. A pie chart describing the percentage of time participants perform spasticity injections under US guidance.

**Fig 3:**
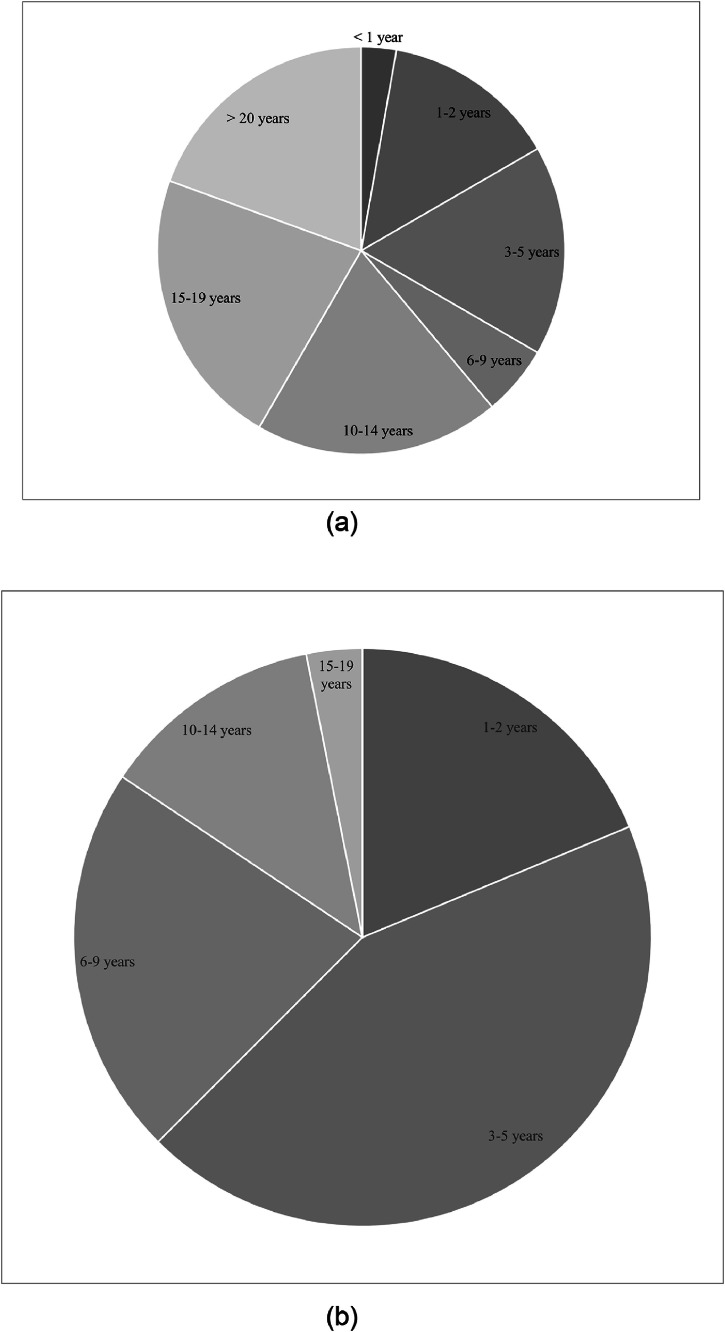
Participants’ years treating spasticity and years using the US in their spasticity practices. A. A pie chart describing the range of participants’ years of clinical experience treating spasticity. B. A pie chart describing the range of participants’ years of using the US for spasticity management.

Although 91% of survey respondents reported using US in their spasticity practice, only 34% indicated that they use this technique for >3-quarters of their spasticity injections. Figure 2 describes the proportion of spasticity injections participants completed with US guidance. Physicians with a wide breadth of experience treating spasticity completed the survey, ranging from 1-2 years (14%) to >20 years (19%). Most respondents (60%) had >10 years of injecting experience. Of those who indicated that they use US for spasticity management, 44% reported that they had been using the technique for 3-5 years. In total, 38% reported that they had been using US for >5 years with 16% indicating >10 years of experience using US for spasticity management. Figure 3 further elaborates on participants’ experiences with spasticity treatment and US. Spearman's rank-order correlation was used to assess the relationship between years of US use and implementation into practice. There was a significant, moderately positive correlation between years of US use for spasticity management and the percentage of spasticity injections performed under US guidance (*r*=0.51; *P*<.05) where physicians with more years of experience treating spasticity with US used US for a higher proportion of their injections.

Although a high percentage of survey respondents reported using US for spasticity management, several physicians indicated that they also employ several other guidance techniques for the delivery of BoNT for spasticity. A total of 77% indicated that they use anatomical landmarks to aid with target identification; 74% use electromyography, 54% use electrical stimulation; and 20% use computed tomography fluoroscopic guidance. US guidance was ranked by participants as the most frequently used guidance technique, followed by electromyography, anatomical landmarks, electrical stimulation, and computed tomography fluoroscopic guidance. Participants could select multiple choices, but the use of two guidance techniques simultaneously, for example US and electromyography, was not a potential option. We do not say or mean to imply that EMG cannot be used simultaneously with US. Table 2 demonstrates the array of guidance techniques that participants use regularly.

**Table 2:** Ranking of most used to least used guidance techniques in spasticity practice

Item	Overall rank	Score	Number of rankings
Ultrasound	1	132	29
Electromyography	2	129	28
Anatomical landmarks	3	123	30
Electrical stimulation	4	96	23
Computed tomography fluoroscopy	5	48	15
Other	6	16	8

Participants were asked to rank the guidance techniques that they used most frequently in their spasticity practices. Participants did not rank techniques that they did not use. When items were ranked as the most used, they received a score of 6, when they were ranked as second received a score of 5, and so on. If items were not ranked, they were assigned a score of 0 for that participant. The most used guidance technique is reported as that with the highest score: ultrasound. Eight participants indicated that they use other guidance techniques but failed to report them.

**Fig 4:**
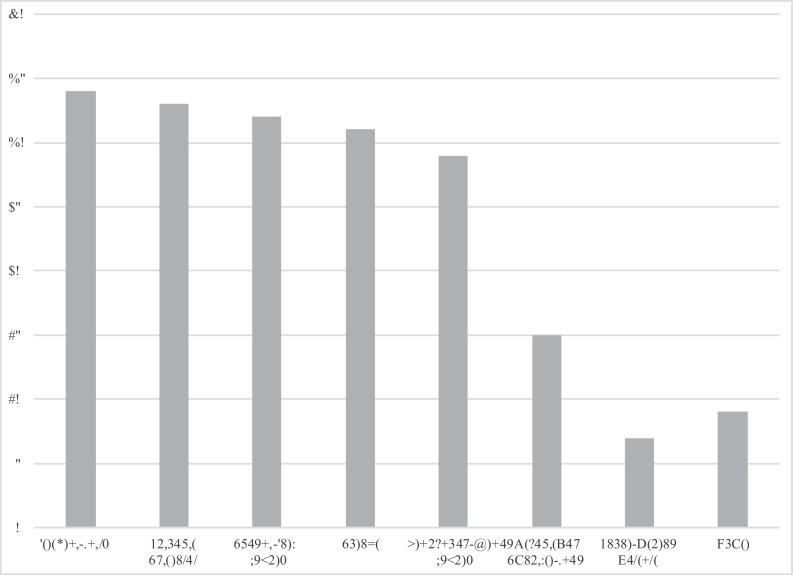
Etiologies treated. The bar graph shows the number of patients presenting with specific etiologies treated by the respondents. “Others” included vascular dementia, brain and spinal cord tumors, Friedreich's ataxia, genetic diseases, hereditary spastic paraparesis, and hemifacial spasm.

Participants reported treating a range of patients, from 0-5 patients per week (33%) to > 20 patients per week (8%). Respondents reported treating various etiologies (refer to fig 4), including both adult and pediatric populations, in a variety of settings; participants treated patients at academic (70% of respondents) and nonacademic centers (77% of respondents).

There are several potential uses for US in spasticity treatment. In total, 91% of survey respondents indicated that they use US for BoNT injections, rendering it the most common application. Other potential uses for US in spasticity treatment include musculoskeletal (MSK) injections (used by 72% of respondents), diagnostic nerve blocks (used by 47% of respondents), MSK condition diagnosis (used by 34% of respondents), and phenol or alcohol denervation (used by 31% of respondents).

**Fig 5:**
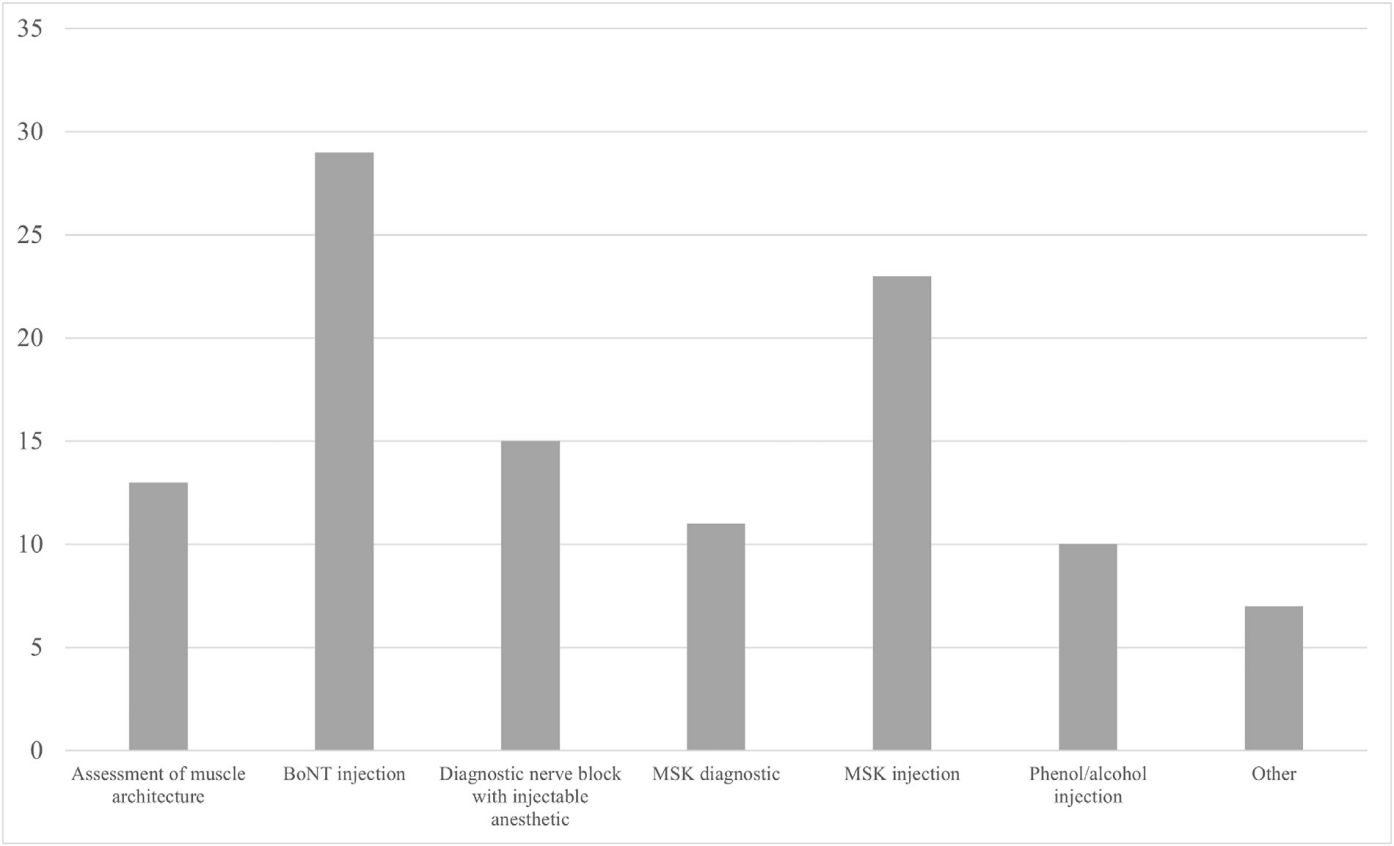
The array of purposes for which participants reported using the US in their spasticity practices. The various purposes that the US was used for in spasticity practice are plotted against the number of physicians who reported using the US for said purposes. Participants were asked to choose all that applied to them. Other purposes were reported as cryoneurolysis (n=3), neuromuscular correlation with electrodiagnostic medicine (n=1), peripheral nerve injections for pain management (n=1), cervical dystonia (n=1), pulsed radiofrequency neurolysis (n=1), and increase the potential effect of toxin when pretreatment goals are not achieved in patients who are not responsive to electrical stimulation (n=1).

The longitudinal assessment of muscle atrophy and echo intensity can be validly tracked using US^11^; however, only 50% of survey respondents reported knowing a validated scale for muscle architecture assessment. Moreover, 41% of survey respondents indicated using the US for muscle architecture assessment. All who indicated that they were familiar with such a scale described it as the modified Heckmatt scale, the Heckmatt scale, or grayscale pixel count. Figure 5 demonstrates the range of purposes for which US is used in clinical practice.

There were several barriers identified that may prevent physicians from using the US, the most impactful of which being clinician time constraints. In total, 65% of participants believed that using US would slow them down compared with using other localization techniques. Physicians felt that time constraints were an important barrier to US implementation. Regardless of years of experience with spasticity treatment and years of experience injecting with US guidance, respondents reported that there was a problematic increase in the time required to complete injections with the US compared with other guidance techniques. Experience also does not seem to influence how highly participants ranked the importance of time constraints as a barrier to implementation relative to other potential barriers. Other important barriers to using US for spasticity management included clinic financial resources, lack of adequate training, lack of physician compensation, or lack of effectiveness (from clinical experience). Barriers are discussed further in table 3.

**Table 3:** Participants’ perceived barriers to implementing US or using US more frequently in their spasticity practices

Item	Overall rank	Score	Number of rankings
Clinician time constraints	1	122	25
Financial – clinic resources	2	95	20
Lack of adequate training	3	79	17
Financial – physician compensation	4	65	18
Lack of effectiveness from clinical experience	5	30	8
No barriers	6	21	5
Lack of evidence in the literature	7	11	4
Financial – patient resources	8	10	3
Other	9	7	2

Participants were asked to rank 6 barriers to implementing US into clinical practice or to using US more frequently in their practice in accordance with which they felt had the greatest effect on them. Participants only ranked their most important 6 barriers. When items were ranked as the most important barrier by participants, they received a score of 6, when items were ranked as the second most important, they received a score of 5, and so on. The score is calculated as the sum of all the individual scores of the participants. The item with the highest score—the most important barrier—was clinician time constraints. Other barriers were reported as difficulties with positioning and clinic space (n=1), and a steep learning curve (n=1).

Nearly half (47%) of the survey respondents indicated that they believed there were “risks” associated with US use. The most frequently cited “risk” was increased time requirements for procedures, which may require uncomfortable patient positioning. Secondary “risks” included a lack of feedback comparable to electrical stimulation, potential infection because of improper cleaning, potential needle-stick injuries while positioning the patient and attempting to align the needle with the target structure, or potential placebo effects from choosing a seemingly more elaborate treatment option.

The majority (76%) of participants reported having training in US for spasticity management. Most commonly, training was accrued during residency by attending physicians, attending annual meetings, or attending industry-sponsored in-person training events. In addition, 70% of respondents reported having some form of training in the US for MSK diagnostic or injection purposes. The sources of training were similar to those used in the US for spasticity training. Almost half (49%) of survey respondents reported that they are involved in US education for spasticity management, either as participants or instructors.

## Discussion

In the present study, 36 Canadian physicians and surgeons were surveyed to provide insights into the landscape of US usage and to identify potential avenues for improvement. Most survey respondents reported using US in spasticity treatment, mostly for BoNT injections but also for MSK injections and diagnosis, phenol and alcohol neurolysis, and muscle architecture assessment. Notably, 2 of the 3 respondents who did not use the US reported lacking access to one. Several barriers (see table 3) were identified that may limit the adoption of US into clinical practice or limit its usage in clinics that already have access to the technology. Physicians’ training was probed, revealing that 3 quarters of respondents had training in US for spasticity management.

BoNT injections were the most frequently reported use of US in spasticity treatment. In this context, the US has documented noninferiority compared with other injection guidance techniques.^6^^,^^7^ As such, US can be used reliably and safely for BoNT injections when adequate training is provided. In contrast with other guidance techniques, US allows for the visualization of structures that surround the target, for example, blood vessels. The accidental puncture of a blood vessel is a possible adverse event that can occur during BoNT injections. As such, an advantage of US in spasticity injections may be that it facilitates procedures by providing a clear understanding of where the needle is relative to blood vessels and nerves. Although BoNT injections are the most reported use of US, respondents indicated using the technique for several other purposes.

In addition to BoNT injections, US is a valid and reliable tool for the longitudinal assessment of muscle echo intensity.^11^ Modified Heckmatt scale assessments can be completed during injection target localization, not increasing the time required per patient. Less than half of the survey respondents indicated that they currently use the US for this purpose in their spasticity treatment. Furthermore, only half of the participants indicated that they were aware of a validated scale for the assessment of muscle architecture. Increasing physicians’ knowledge of the importance of muscle architecture in spasticity management and assessment techniques could be of value in improving the standard of care for patients with spasticity. Furthermore, because muscle echo intensity has been shown to influence spasticity treatment response,^12^, ^13^, ^14^, ^15^ we argue that knowledge of such assessment tools is crucial to allowing for individualized treatment decisions. This aspect of US usage could be incorporated into future US for spasticity teaching efforts to improve clinical decision-making, especially among physicians with less experience treating spasticity or using the US.

Several potential barriers to incorporating the US into spasticity practice were identified by survey respondents. In all, 44% of participants reported a lack of adequate training with US as a barrier to implementing US into clinical practice. Although most respondents reported having attended some training for US-guided injecting, there was notable heterogeneity in the source and structure of this training. These responses raise important concerns regarding the length, quality, and intensity of the training received, and suggest a need for revisiting and standardizing the currently offered curricula. As outlined by Alter and Karp^5^, the training required to become proficient in US guidance for BoNT procedures requires a commitment to months of courses, readings, self-study, scanning, and hands-on practice.Click or tap here to enter text. Future research should investigate the standardization and implementation of US training into the curriculum of trainees who will treat spasticity, as well as with practicing physicians looking to improve or add the technique to their toolkit.

A significant barrier to US implementation in clinical practice was reported to be time constraints on the physician and the clinic. Survey respondents felt that US-guided injections took longer than injections with other guidance techniques. The increased time requirement for US compared with other localization techniques is an important factor to consider when choosing an appropriate guidance technique for BoNT injections for each patient. To our knowledge, differences in injection time requirements between guidance techniques have not been studied. Amongst participant to this study, experienced physicians still cited time as a concern. However, they may be perpetuating poor technique. We suspect that specific training to improve physicians' scanning and injection technique could improve efficiency, but study would be needed to prove efficacy of an education intervention. More than half of the physicians and surgeons polled reported that there is no additional specific remuneration for BoNT injections under US guidance in their province or territory. A total of 70% of these physicians agreed that remuneration would increase the use of US. Therefore, specific remuneration for US-guided BoNT injections and the adoption of the procedure should be explored further. We postulate that if the goal is to increase the number of proceduralists using US guidance, it may be necessary to consider additional specific remuneration. However, it would be vital to ensure that the level of training is adequate, because it may be detrimental to have undertrained physicians using US guidance instead of alternative guidance techniques.

Approximately half of the survey respondents reported that they believe there are “risks” associated with US usage in spasticity management. Many respondents indicated that increased time requirements for US-guided injections were not only barriers but also a risk to the patient and the injector. They believed that increasing the time for treatments would present a risk to the patient because they may be required to maintain an uncomfortable position for a prolonged period, and that this could also present a risk to the injector because ergonomics may be compromised. Research investigating the optimization of injection ergonomics for ultrasound injections has been published recently.^17^ Future educational efforts should include specific sessions for patient positioning to maximize patient comfort while maintaining ideal ergonomics for proceduralists. Additionally, future educational efforts should discuss strategies to make US-guided injections and assessments as efficient as possible.

There were several other “risks” reported that we argue are not specific to US usage or are not reasonable. For instance, respondents reported an increased risk of incurring needle-stick injuries while performing injections under US guidance. Although this is a risk with all spasticity injections, we would speculate that the use of US would not make these types of injuries occur more frequently; further study is required. In addition, several respondents reported risks related to the chance of acquiring an infection from US gel contamination. While performing US-guided spasticity injections, it is important to use sterile gel. The risk of US gel contamination leading to an infection is nullified if the sterile gel is used. Moreover, the risk of infection from improper cleaning was reported. Although this risk is not exclusive to US-guided injections, it is vital to ensure that the US probe is cleaned properly or to use a sterile probe cover before being used on any patient. Proper US probe cleaning and maintenance techniques should be incorporated in future training sessions.

Another “risk” identified was the chance that the procedure could produce more of a placebo effect than injections with traditional guidance techniques. Although it is possible that such an effect could exist, such an effect would have likely been identified in studies aimed at assessing the efficacy of alternative guidance effects. These studies have demonstrated noninferiority for the US compared with alternatives.^6^^,^^7^ Although these studies were not powered to detect equivalence for US compared with e-stim guidance, the authors did not notice or discuss the presence of any placebo effect. Furthermore, if a placebo effect were present, it would not necessarily be a risk to the treatment but may result in an increased response magnitude, which would benefit patients.

### Future directions

This cross-sectional survey has described how Canadian physicians and surgeons are using the US in their spasticity practice. At least 34% of the Canadian colleagues used US guidance in >75% of their spasticity injections. Future studies should therefore better investigate strategies to reduce barriers that may affect the utilization of US in a busy spasticity practice. In addition, future cross-sectional surveys should be developed to analyze the content of residency training programs for US in spasticity. This will help develop research focused on strategies to implement robust teaching curricula to improve clinical teaching practices and improve the quality and standardization of training (content and quality of teaching) received in residency or medical school. Furthermore, future studies, using cross-sectional surveys, should assess the adoption of US in spasticity practice in other countries and aim to assess the confidence levels of physicians using US to understand the quality of training and teaching programs. Almost half of the survey participants indicated that they are currently involved in teaching or training efforts for spasticity treatment with the US. When asked about what they would like to see more of in the future, many participants indicated that they would appreciate more training events. There are several resources available to physicians and residents looking to improve their US skills or introduce US into their spasticity practice. Some examples of platforms with high-quality educational content available are CANOSC, Skills, Training, Evaluation and Performance (STEP), and Toxin Academy.^18^, ^19^, ^20^ Further efforts should be dedicated to increasing awareness of these programs and expanding their content to improve the adoption of ultrasound in spasticity practice, including a hands-on component to the curricula. A future study is also needed on echotexture and the use of the US to guide treatment.

### Limitations

This survey was sent from the CANOSC mailing list. Physicians who have subscribed to these updates may be more likely to be interested in US for spasticity because CANOSC provides many educational opportunities in this area, potentially leading to some sampling bias. Our response rate was also small, limiting statistical operations and generalizability. Future studies should aim to include a larger sample to further test hypotheses generated from the present work. Lastly, a very small number of survey respondents indicated that they did not use US in their spasticity practice. This poor representation could be in part because of the topic of the survey; physicians who are not actively using US or who are not interested in using US may have been less likely to respond to the survey. Lastly, Canada is a developed country with access to many resources that may be unavailable in certain areas of the world. As such, these findings should not be generalized internationally.

## Conclusions

US is a useful tool in spasticity management that is widely used for localization in our sample. Several avenues for future teaching efforts have been identified by this survey. Important avenues for future teaching include addressing perceived risks such as increased time expenditure, risk of infection, and poor patient positioning and injector ergonomics. Additionally, the use of US as a tool in muscle architecture should be included at training events. Systemic efforts should be considered to increase the quality and standardization of training for the use of US in spasticity management.
